# Attitude Angle Compensation for a Synchronous Acquisition Method Based on an MEMS Sensor

**DOI:** 10.3390/s19030483

**Published:** 2019-01-24

**Authors:** Huanhuan Tian, Yixiao Liu, Jiqin Zhou, Ying Wang, Jing Wang, Weigong Zhang

**Affiliations:** 1 College of Information Engineering, Capital Normal University, Beijing 100048, China; entele_thh@163.com (H.T.); yixiao.liu@cnu.edu.cn (Y.L.); zhoujiqin@cnu.edu.cn (J.Z.); 2 Beijing Advanced Innovation Center for Image Theory and Technology, Capital Normal University, Beijing 100048, China; wangyingstudio@163.com (Y.W.); jwang@cnu.edu.cn (J.W.); 3 State Key Laboratory of Computer Architecture, Institute of Computing Technology, Chinese Academy of Sciences, Beijing 100048, China; 4 Beijing Engineering Research Center of High Reliable Embedded System, Capital Normal University, Beijing 100048, China

**Keywords:** Quartz Vibrating Beam Accelerometer, attitude angle compensation, strapdown inertial navigation system, improved equal-precision frequency measurement, FPU

## Abstract

As a new type of micro-electro-mechanical systems (MEMS) inertial sensor, the Quartz Vibrating Beam Accelerometer (QVBA) is widely used in intelligent sweeping robots, small aircraft, navigation systems, etc. For these applications, correcting and compensating the attitude angle with the result of acceleration plays an important role to improve the measurement accuracy. The synchronization error between the measurement of the accelerometer and gyroscope attitude angle has an adverse impact on the accuracy of the attitude angle. In this paper, a synchronous acquisition scheme of the accelerometer and gyroscope attitude angle in a strapdown inertial navigation system (SINS) is proposed. At the same time, to improve the sampling accuracy and the conversion speed of QVBA, an improved equal-precision frequency measuring method is also implemented in this paper. The hardware float point unit (FPU) is used to accelerate the calculation of the frequency measurement value. The long-term cumulative error of the frequency measurement value is less than $10^{-4}$. The calculation process time from sampling to attitude angle compensation calculation is reduced by 40.8%. This work has played a very good role in improving the measurement accuracy and speed of the SINS.

## 1. Introduction

With the development of micro-electro-mechanical systems (MEMSs) technology, the Quartz Vibrating Beam Accelerometers (QVBAs), a new type of MEMS inertial sensor with full digital pulse output, are drawing increasing attention in small aircraft, robots, navigation systems, augmented reality systems, and so on due to the advantages of high accuracy, small size, and low cost [1]. In the strapdown inertial navigation system (SINS), QVBAs are mainly used to measure the linear acceleration of the body. Because their good static characteristics, the acceleration value can be accurately measured in the static state. However, during flight, they are susceptible to temperature and noise because of the poor dynamic characteristics. To solve the accuracy problem of accelerometer measurement, many high-precision inertial navigation systems introduce a strapdown mechanism with the integration of an accelerometer and gyroscope. In these strapdown inertial navigation systems, a gyroscope is applied to measure the rotational angular velocity with its good tracking performance and good dynamic response characteristics, which makes up for the disadvantage of the accelerometer. Then, through the fusion of these two kinds of data, a high-precision attitude angle can be obtained.

However, these strapdown mechanisms present new challenges in terms of how to integrate the data of the accelerometer and gyroscope to achieve high attitude accuracy. Many state-of-the-art research works handle this problem from the point of optimizing the data fusion algorithms. In Refs. [2,3], the Kalman filter was used to fuse the data of the accelerometer and gyro, and on this basis, the random drift error of the gyro output angular velocity was corrected to improve the attitude accuracy of the aircraft. As the Kalman filter can only be used in the linear system under the noise of Gaussian white noise, Ref. [4] proposed an extended Kalman filter (EKF), which extends the Kalman filter theory in the nonlinear field. In Refs. [5,6], the EKF was adopted as the fusion algorithm of attitude estimation, but only in the weak nonlinear system. In Ref. [7], a new mixed Kalman filter based on extended and unscented Kalman filter was designed to estimate attitude angles by a sensor embedded on the projectile. In Refs. [8,9], the particle filter was used as the attitude estimation algorithm, which solved the nonlinear and non-white noise limits of the systems and improved the filtering precision. However, the particle filter also has a heavy calculation burden and is not suitable for a low-cost navigation system, so in Refs. [10,11], the complementary filter algorithms in Euler angles and direction cosines were given respectively. In Refs. [12,13], a complementary filter based on the quaternion method was presented, which reduced the computational complexity of the attitude solution and improved the efficiency and accuracy. Simple and yet practical linear complementary filters have been successfully used in practical applications. For example, Ref. [14] used small rotational motions, where the angular velocity was used to complement the vector measurements to improve the estimation accuracy through an appropriate filtering. In Ref. [15], a technique for estimating the angular velocity of a rigid body from a single vector measurements was proposed. It did not use attitude information or rate gyroscopes as inputs. Instead, vector measurements were filtered directly through a nonlinear observer estimating the angular velocity. Euston et al. [16] presented a nonlinear quaternion-based complementary filter to estimate the attitude of an unmanned aerial vehicle (UAV) given measurements from a low-cost IMU. The filter was augmented by a first-order model of the vehicle dynamics to compensate for external centripetal acceleration. In UAV applications, the use of complementary filters [16,17,18] is often preferred to the EKF because EKFs can be complicated to implement and the convergence is slower because of the time required for the linear regression iterations. 

As an important process of the SINS, optimizing the fusion algorithms can guarantee higher attitude accuracy in principle. However, simply improving the fusion algorithms is not sufficient to attain the optimal result. This is because no matter what the fusion algorithm is, the degree of synchronization between the measurement of acceleration and gyroscope attitude angle has a very serious impact on the accuracy of compensation and correction, thus affecting the accuracy of attitude. However, in the current SINS, accelerometer and gyroscope work independently under their respective sampling frequencies. There is a degree of asynchrony between them that leads to a certain error, so asynchronous acquisition will cause a large cumulative error. It has an adverse impact on the accuracy of the final calculated attitude angle and even causes the data to be inaccurate. So, synchronization acquisition is also necessary to enhance the accuracy of the attitude angle. However, to the best of our knowledge, no work considers synchronization problems of the accelerometer and gyroscope.

In addition, in the SINS, the accuracy of inertial instruments themselves, such as the accelerometer and gyroscope, is the basis of the system accuracy. There are some recent works that have attempted to improve the accuracy of inertial instruments. In Ref. [19], the structural process and processing technology of the instrument were studied to improve the measurement accuracy of the instrument and the accuracy of the inertial navigation system. Since this method was fulfilled through hardware, it increases the overall cost. In Ref. [20], the instrument or system was tested and modeled before the actual application, thus the accuracy of the instrument was improved by error compensation. This method needed no additional hardware overhead. However, it did not suit the occasion of correcting the dynamically changing error signal.

To address the above problems of the SINS, in this paper, we propose a new method aiming at providing a more accurate attitude angle with low cost and high efficiency. This paper makes two principal contributions:

Firstly, we propose a synchronous data acquisition mechanism that synchronizes the sampled signals of accelerometers and gyroscopes. The key idea of our design is to synchronize the operation of the accelerometer and gyroscope before the execution of the fusion algorithm. This method eliminates the asynchronous error caused by the accelerometer and the gyroscope working independently under their respective sampling frequencies. On this basis, the two kinds of data are fused by the complementary filtering algorithm based on the quaternion method presented in Ref. [11], so that the attitude angle is corrected and compensated during the acceleration change, which effectively improves the measurement accuracy.

The second principal contribution of the paper is to propose an improved equal-precision frequency measurement method to measure the frequency of the accelerometer with high precision, which improves the measurement accuracy from the point of the accelerometer itself. 

In addition, to improve the conversion speed of the quartz vibrating beam accelerometer, we introduce a hardware-based FPU to accelerate frequency measurement.

In the experiment section of the paper, we evaluate the validity of our design approach and compare it with the state-of-the-art approach for the improvement of attitude accuracy. We implement multiple sets of experiments in an SINS. The results show that the long-term cumulative error of the frequency measurement value is less than $10^{-4}$, and the calculation process time from the sampling to the attitude angle compensation calculation is reduced by 40.8% in this system. Moreover, with our proposed synchronous data acquisition mechanism, it significantly improves the accuracy of attitude angle output.

The rest of this paper is organized as follows. Section 2 introduces the background and motivation of the work. In Section 3, the synchronous data acquisition mechanism of the accelerometer and gyroscope, the improved equal-precision frequency measurement method, and the FPU are designed and analyzed in detail. In Section 4, the effectiveness of the proposed method is verified by experiments. Section 5 gives the conclusions.

## 2. Background and Motivation

In this section, we first introduce the operating principle of QVBA. Then, we illustrate the equal-precision frequency measuring technology. After that, we describe the architecture of SINS. Finally, we outline the motivations of our work. 

### 2.1. Operating Principle of QVBA

QVBA [21] is a new kind of high precision solid state sensor based on the force-frequency characteristics of a quartz vibrating beam. It is based on the piezoelectric effect and the force-frequency characteristic of the quartz resonator. Its internal alternating electric field makes two push-pull installed quartz beams vibrate in a stretching mode. When the accelerometer is subjected to external acceleration, the sensitive mass generates one inertial force to act on these two quartz beams, respectively. One of the quartz beams is subjected to pressure and its resonant frequency will reduce; while the other one is subjected to a tensile force, its resonant frequency will increase. The frequency difference of the final two quartz beams is proportional to the externally applied force, that is, which is proportional to the acceleration, and then the value of the acceleration is measured [22]. Accordingly, the frequency measurement accuracy directly reflects the accuracy of the acceleration measurement.

As mentioned above, each accelerometer will output two frequency signals. The difference between these two frequency signals and its relation to the acceleration can be calculated as follows:

(1) $$f_{2}-f_{1}=L_{1}(L_{0}+a+L_{2}a^{2})$$

$L_{0}$ is the zero offset value, $L_{1}$ is the scale factor, and $L_{2}$ is the second order nonlinear coefficient. $f_{1}$ and $f_{2}$ denote the output of the sensitive quartz beam, $F_{1}$ and $F_{2}$, respectively, the unit is Hz, and a denotes the immediate acceleration, the unit is g.

### 2.2. Equal-precision Frequency Measurement Method

The commonly used frequency measurement methods are periodic frequency measurement, direct frequency measurement and equal-precision frequency measurement. The periodic frequency measurement method obtains the frequency by counting pulses of the standard frequency signal within one cycle of the signal to be measured. The direct frequency measurement method obtains the frequency by counting pulses of the signal to be measured within a given gate time [23,24]. The advantages of these two methods are that the principle is simple and easy to implement. However, in the measuring process of these two methods, count errors are inevitable due to their counting principles and the measurement accuracy varies with the frequency of the measured signal. Through error analysis, the periodic frequency measurement method is suitable for the measurement of low-frequency signals, and the direct frequency measurement method is suitable for the measurement of high-frequency signals. Neither of them can meet the measurement accuracy of both high and low frequency signals. Unlike the previous two methods, the measurement accuracy of the equal-precision frequency measurement method is irrelevant to the frequency of the measured signal. It not only provides high measurement accuracy, but also maintains constant measurement accuracy for the whole range of measurement frequency [25].

### 2.3. Architecture of Strapdown Inertial Navigation System

**Figure 1:**
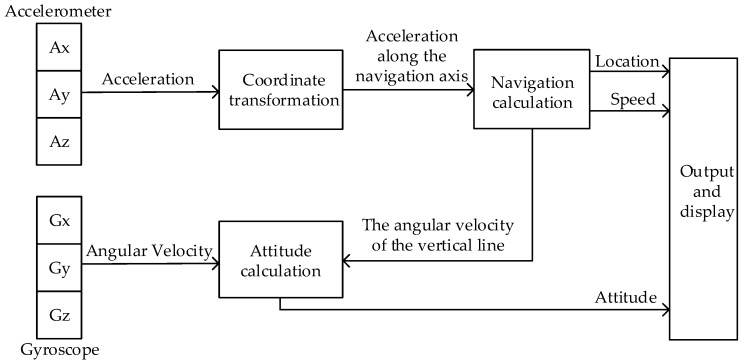
The schematic diagram of the strapdown inertial navigation system.

The SINS is a frameless system, which consists of three accelerometers, three gyroscopes, and a microcomputer. Figure 1 shows the schematic diagram of a typical SINS. In this system, accelerometers and gyroscopes are directly mounted on the carrier to measure acceleration and angular velocity, respectively. The microcomputer is used to calculate the attitude matrix in real time. In this way, the accelerometer information of the carrier coordinate system is converted into the information under the navigation coordinate system. On this basis, the navigation calculation is performed, and finally the speed, position, and attitude information of the carrier are obtained.

### 2.4. Motivations

While providing long-term high accuracy is important for many navigation applications, such as spacecrafts, missiles, aircrafts, and warships, existing SINS do not effectively and efficiently support real-time acquisition of high accuracy attitude. First, the SINS do not provide synchronous sampling of the accelerometers and gyroscopes, so there will be time deviations between these two kinds of data after a period of time. Since the final output attitude is the fusion of these two kinds of data, their time difference results in inaccuracy of the calculated attitude angle. Therefore, proposing a synchronous acquisition scheme of the accelerometer and gyroscope is necessary to correct and compensate the attitude angle for long-term navigation, especially if the acceleration changes. This work focuses on an efficient synchronization mechanism of SINS to improve the accuracy of the calculated attitude angle.

In addition, it is an important way to correct and compensate the attitude angle by improving the measurement accuracy of the accelerometers or gyroscopes. As described in Section 2.2, the equal-precision frequency measurement method can provide high measurement accuracy. However, this method still has a problem of differentiating a pulse count error when counting standard signals. So, to improve the sampling accuracy of QVBA, this work proposes an improved equal-precision frequency measurement method.

The floating point number has the characteristics of high precision, wide data representation range, etc., thus it is applied in QVBAs during the frequency calculation. FPU can complete the floating point operation with high operation speed [26]. Considering the inefficient conversion speed of the existing QVBAs, a hardware FPU is introduced in the frequency measurement module to accelerate the calculation of the frequency value.

## 3. Proposed Scheme

### 3.1. Framework Overview

**Figure 2:**
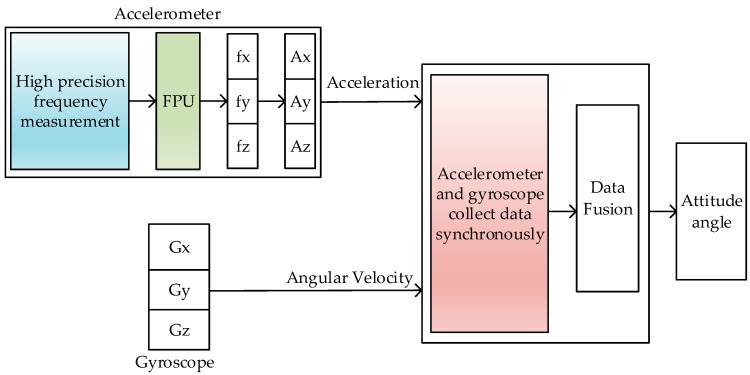
The block diagram of our work.

We propose an attitude accuracy improvement framework to solve the above problems. An overview of our scheme is illustrated in Figure 2. It consists of three components: A synchronous data acquisition module is designed to fulfill the synchronous sampling of accelerometers and gyroscopes. It solves the accuracy problem of attitude angle correction and compensation to improve the accuracy of the attitude angle calculated in the SINS; an improved equal-precision frequency measurement module is proposed to improve the sampling accuracy of the accelerometer. To improve the conversion speed of the QVBA, a hardware FPU is introduced, which speeds up the calculation of the frequency measurement value.

As shown in Figure 2, in the SINS we designed, the synchronous acquisition scheme, high-accuracy data acquisition of accelerometer, and FPU are the focus of this paper. The design of the gyroscope data acquisition module and data fusion algorithm refer to the existing methods. The basic working principle of these modules are as follows. The navigation computer counts the demodulation pulses of the gyroscope and the buffeting pulses of the gyroscope. Once the buffeting pulses are inputted into the filter circuit through the input filter circuit, they are sent to the FPGA through the Schmitt trigger shaping circuit. The demodulation pulses are generated by the gyroscope demodulation circuit and sent to the counter. At this time, the sampling signals of the accelerometer and the gyroscope can be synchronously latched. Then, based on the data synchronous of the accelerometer and gyroscope, we use the quaternion method for attitude calculation and use the complementary filtering method for data fusion. The specific implementation can be found in Ref. [11]. 

### 3.2. Synchronous Acquisition Mechanism

**Figure 3:**
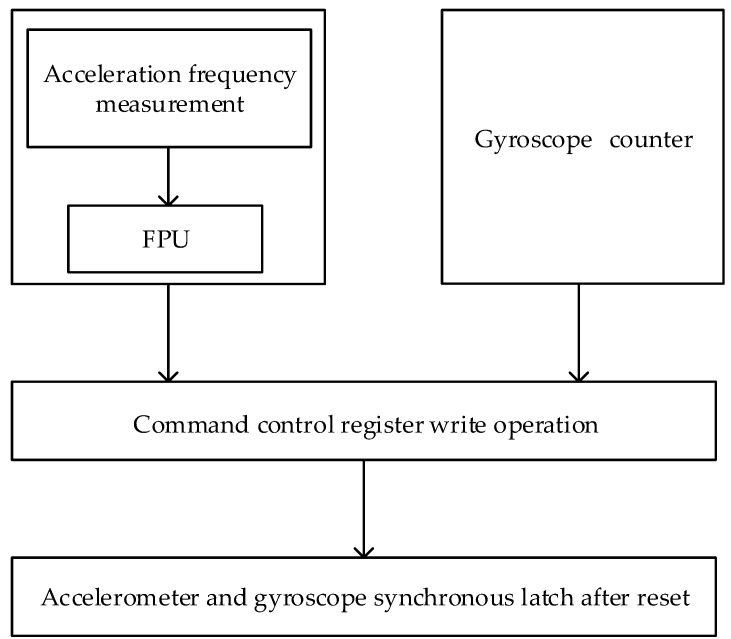
Hardware design of the synchronous data acquisition module.

The design of the synchronous data acquisition module is divided into two parts, including hardware and software. The implementation process is as follows: 

The hardware design is shown in Figure 3. We implemented the hardware part on a FPGA chip using the very high-speed integrated circuit hardware description language (VHDL). Since there are three accelerometers in the SINS, the designed frequency sampling system can measure the frequency signals of three accelerometers. After obtaining the measured value, a signal of the completion of the counting value lock is generated, and the floating point calculation module is notified to read the counting value for frequency calculation. At the same time, the navigation computer counts the demodulation pulses of the gyroscope and the buffeting pulses of the gyroscope. Once the buffeting pulses are inputted into the filter circuit through the input filter circuit, they are sent to the FPGA through the Schmitt trigger shaping circuit. The demodulation pulses are generated by the gyroscope demodulation circuit and sent to the counter. At this time, the sampling signals of the accelerometer and the gyroscope can be synchronously latched through write operations performed on a command control register.

**Figure 4:**
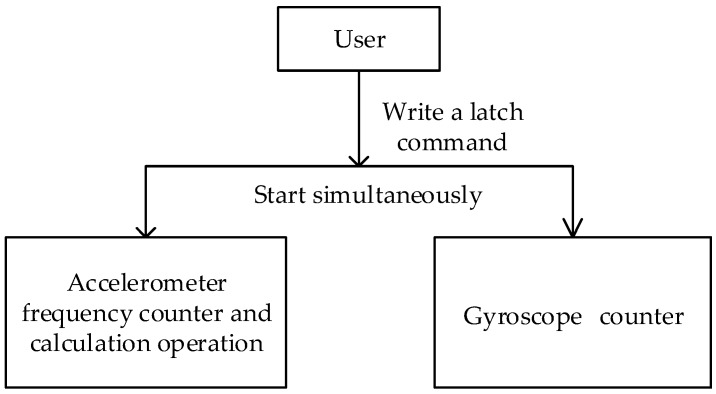
Software design of the synchronous data acquisition module.

Figure 4 demonstrates the software design. We implemented the software part on a DSP chip using C language. In the synchronous data collection mode, when the user writes a lock command to the gyroscope counter, the lock operation and calculation operation of the accelerometer frequency measurement counter are also started simultaneously. To ensure the readout reliability of these two counters, the measured value cannot be read directly. When a counter needs to be read, the software should first send a latch command by writing to the corresponding counter latch command register. Then, the current count value of the gyroscope counter is latched into the count value latch register, meanwhile, the accelerometer frequency measurement value is also latched into the count value latch register. So, the count value of each counter can be obtained by reading the count value latch register. This method also ensures that the count value of the gyroscope and the acceleration can be latched synchronously, thus the errors caused by the software reading different counters and frequency values serially can be eliminated.

### 3.3. Improved Equal-precision Frequency Measurement Method

**Figure 5:**
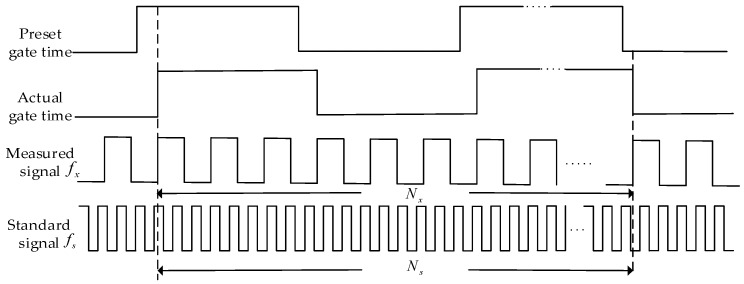
The time diagram of the equal-precision frequency measurement method.

In equal-precision frequency measurement, two counters and a known standard frequency signal are required. The principle of frequency measurement is to give the preset gate opening time first. At this time, the counter does not start counting. However, when the rising edge of the measured signal arrives, the synchronization signal is used to synchronize the gate time signal and the measured signal. At the same time, the counter starts counting. At this point, the count value of two counters will be obtained, and the frequency value of the measured signal can be obtained by combining with the frequency value of the standard signal. Figure 5 shows the time diagram of the equal-precision measurement method; the counter only calculates the number of pulses in the actual gate width time.

It can be seen from Figure 5 that the actual gate time is not a fixed value, but an integral multiple of the signal period being measured. Therefore, there is no counting error when counting the tested signal. However, the actual gate time signal is not synchronized with the standard signal, so there still exists the error problem of one pulse difference when counting the standard signal.

Suppose that in a gate time, *T*, the counter count value, *N_x_* denotes the number of pulses of the signal to be measured, *N_s_* denotes the number of pulses of the standard signal, and the frequency of the standard signal is *f_s_*. The frequency of the measured signal is *f_x_*, then there are:

(2) $$N_{x}=f_{x}\times T$$

(3) $$N_{s}=f_{s}\times T$$

Thus, the frequency value of the measured signal is:

(4) $$f_{x}=\frac{N_{x}}{N_{s}}\times f_{s}$$

**Figure 6:**
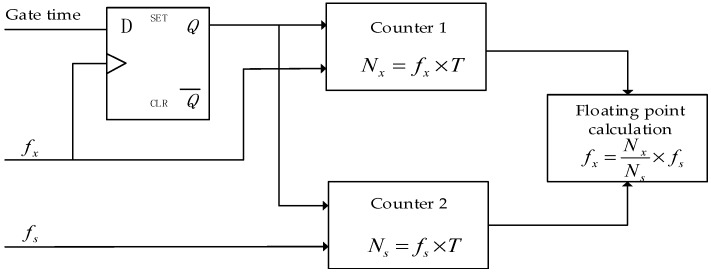
Architecture of the equal-precision frequency measurement method.

The improved equal-precision frequency measurement method uses a standard frequency signal, *f_s_*, to measure the measured frequency signal, *f_x_*, that reduces the error caused by the standard signal pulse count during the measurement. The specific implementation and working process is as follows:

As shown in Figure 6, Counter1 and Counter2 are two non-return zero and controllable counters. The standard frequency signal is input from the clock input terminal of Counter1; the measured signal after shaping is input from the clock input terminal of Counter2. When the preset gating signal is at a high level, the rising edge of the measured signal after shaping will start Counter1 and Counter2 simultaneously through the Q terminal of the D trigger, and the count values are *N_x_* and *N_s_*, respectively. When the preset gating signal is at a low level, the rising edge of the subsequent measured signal will cause both counters to be turned off simultaneously.

In a gate time, *T*, two non-return zero counters, Counter1 and Counter2, are used to perform continuous counting in the measurement module. So, the number of pulses, *N_x_*, of the measured signal and the number of pulses, *N_s_*, of the standard frequency are continuously counted. These two counters are all incremented by 1 on the rising edge of the frequency signal. When each counter reaches the maximum value, it automatically rolls back to zero and counts again. If the count value need to be latched, only the current values of the two counters are latched. Since in this situation, the counters are not cleared, the continuity of the counters can be ensured. Moreover, the measurement errors through long-term integration can be eliminated more conveniently. After that, the current latched value subtracts the previous latched value to obtain the count value between the current latch pulse and the last latch pulse.

Compared with the conventional equal-precision measurement method using a periodic gate for data latching, our frequency measurement module uses an active latch signal to latch the counter. The rising edge of the latch pulse is used to set the count value acquisition flag when the pulse arrives. Once this flag is valid, the count values of the standard frequency signal and the measured signal are both latched in the next cycle after the rising edge of the measured signal. This ensures time determinability of the latched count value and synchronicity to the task cycle. After the latch command is synchronized with the rising edge of the measured frequency signal, the current count values of Counter1 and Counter2 are latched at the next rising edge of the standard clock. Then, by subtracting the previous latched value, the count values, *N_x_* and *N_s_*, between adjacent latched commands can be obtained. Equation (4) is the frequency value of the measured signal. 

After *N_x_* and *N_s_* are obtained, a latching completion signal is generated to inform the floating-point calculation module and software for the following frequency calculation. 

### 3.4. FPU 

**Figure 7:**
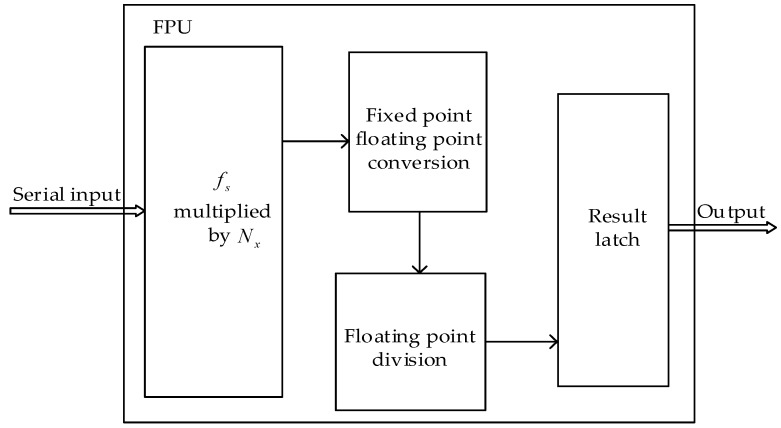
Floating point calculation process.

In this paper, we adopted serial floating-point calculation with a period of 16 µs for the frequency measurement module. Compared to the parallel floating-point calculation, serial one has a low hardware overhead. As mentioned in Section 3.3, the frequency value of the measured signal is calculated according to Equation (4), and the final result is a normalized double precision floating point number that satisfies the IEEE 754 standard. To fulfill this floating-point calculation, our designed FPU is as shown in Figure 7. The FPU includes four processes: Multiplication of fs and *N_x_*, conversion of fixed-point to floating-point, floating-point division, and result latching. Floating point calculations are performed in the order of X1, X2, X3, Y1, Y2, Y3, Z1, Z2, and Z3. A floating-point calculation process starts immediately after the corresponding counter value lock is completed. 

## 4. Experiments and Discussions

### 4.1. Accelerometer Frequency Sampling system

As described in Section 2, SINS requires three accelerometers. Each accelerometer outputs three-channel frequency signals, *f*_1_, *f*_2_, and *f*_3_, among which *f*_1_ and *f*_2_ are frequency signals and *f*_3_ is a temperature and frequency signal, denoting the internal temperature of the accelerometer. Therefore, the frequency sampling system designed in this paper needs to measure and calculate nine-channel frequency signals. Then, based on the previously mentioned Equation (1), we can compute the instantaneous acceleration value according to the frequency values outputted from the accelerometer.

**Figure 8:**
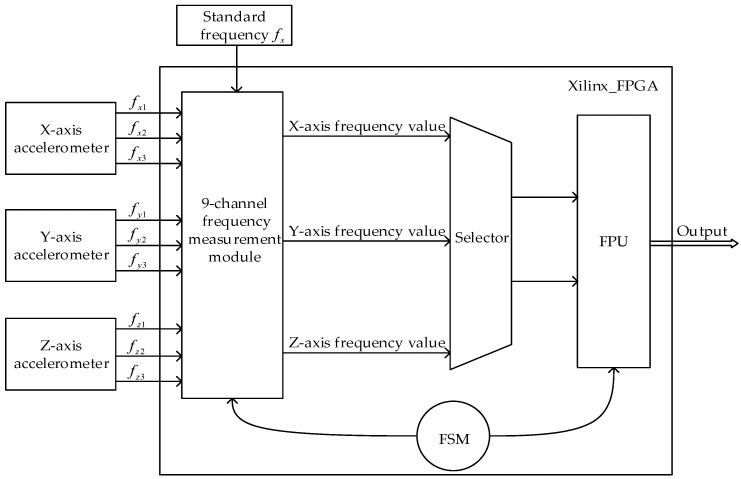
Quartz Vibrating beam accelerometer output and acquisition system.

Figure 8 is the basic structure diagram of the accelerometer frequency sampling system, which is mainly completed in a Xilinx Spartan-3A FPGA chip XC3S1400AN. Among them, the nine-channel frequency measurement module is made up of FPGA logic processing functions using VHDL. It is actually composed of nine equal-precision frequency measurement modules. Every frequency measurement module uses the accelerometer sampling signal and the gyro sampling signal to synchronize their data acquisition. This enables the real-time data fusion of the accelerometer and the gyroscope in the SINS, thus making the output attitude angle more accurate.

### 4.2. Frequency Measurement Error Analysis 

According to the measurement principle, the measurement error of the method for calculating the equivalent precision frequency measurement is analyzed as follows. 

First, the frequency measurement error can be obtained by differentiating Equation (4), as follows:

(5) $$df_{x}=\frac{f_{s}}{N_{s}}dN_{x}-\frac{N_{x}}{N_{s}^{2}}f_{s}dN_{s}+\frac{N_{x}}{N_{s}}df_{s}$$

According to the above principle analysis, it can be seen that in a gate time, the gate time is an integer multiple of *f_x_*, then there is no counting error for the count value *N_x_* of *f_x_* in this time, so $dN_{x}=0$. The error of the count value, *N_s_*, for fs is different by at most one pulse, that is $dN_{s}=\pm 1$.

Then, the relative error of this measurement method can be calculated as:

(6) $$\delta =\frac{df_{x}}{f_{x}}=\frac{df_{s}}{f_{s}}\pm \frac{1}{N_{s}}$$

In Equation (6) above, the frequency measured error is only related to the frequency and count value of the standard signal counter, but independent of the frequency signal to be measured, i.e., the equal-accuracy measurement in the measured frequency band is achieved. The frequency error of the standard signal is Δ*f_s_*/*f_s_*. Due to the high stability of the crystal, the relative error of the standard signal is small and negligible.

If the actual frequency of the measured signal is set as $f_{x}^{\prime }$, another expression of measurement error is given by:

(7) $$\delta =\frac{\left|f_{x}^{\prime }-f_{x}\right|}{f_{x}}\times 100\%$$

If the frequency error of the standard signal is ignored, then according to Equation (7), the actual frequency of the measured signal can be expressed as:

(8) $$f_{x}^{\prime }=\frac{N_{x}}{N_{s}\pm dN_{s}}f_{s}$$

Substituting Equations (4) and (8) into Equation (7), thus:

(9) $$\delta =\frac{dN_{s}}{N_{s}}\times 100\%\leq \frac{1}{N_{s}}=\frac{1}{Tf_{s}}$$

According to Equations (9) and (6), it can be seen that the longer the gate time, *T*, is, or the higher the standard signal frequency, *f_s_*, is, the smaller the relative error of the frequency measurement will be. Assuming that the frequency of the standard signal is 100 MHz, as long as the actual gate time is greater than or equal to 1 s, the maximum relative error of measurement can be less than or equal to $10^{-8}$.

### 4.3. Complementary Filtering Algorithm

**Figure 9:**
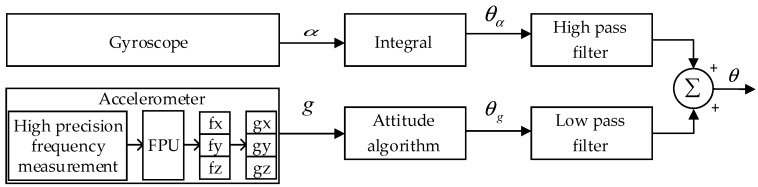
Complementary filtering principle diagram.

We used the existing complementary filtering algorithms [11] for data fusion. The principle of the complementary filtering algorithm is shown in Figure 9. The data measured by the gyroscope is the angular velocity, *α*, and the attitude angle, *θ_α_*, can be calculated by integral operation. As introduced in Section 2.1, the data measured by the accelerometer is three-axis frequency (*f_x_*, *f_y_*, *f_z_*), which is proportional to the externally applied force. Then, according to the Equation (1) in Section 2.1, the three-axis acceleration (*g_x_*, *g_y_*, *g_z_*) can be obtained, respectively. The attitude angle, *θ_g_*, can be calculated by the attitude algorithm. Within a short period of time, the attitude angle acquired by the gyroscope would not be affected by the acceleration of the carrier, so it is accurate. However, the integral drift and temperature drift have a growing influence over time, which makes the result of the gyroscope inaccurate. The average angle measured by the accelerometer is used to calibrate the attitude angle measured by the gyroscope. Therefore, in the short term, the gyroscope is mainly used, and the signal obtained by the gyroscope needs to filter the low frequency part. In the long term, the accelerometer is mainly used, and the accelerometer signal needs to filter the high frequency part. The signals of two complementary bands are added to obtain the calibrated attitude angle, *θ*.

Suppose that the original signal passes through the filter undamaged, the high-frequency noise, *θ_g_*, passes through the low-pass filter, and the low-frequency interference, *θ_α_*, passes through the high-frequency filter. Under the premise of reasonably setting the high/low-pass threshold, the error can be minimized and the true attitude value is approximated by the following calculation formula:

(10) $$\theta =\frac{\tau }{\tau +dt}(\theta^{\prime }+\alpha *dt)+\frac{dt}{\tau +dt}\theta_{g}$$

where *τ* is the time constant, dt is the sampling period, *θ′* is the angle calculated value of the previous period, *α* is the angular velocity measured by the gyroscope, and *θ_g_* is the angle value measured by the accelerometer.

### 4.4. Experimental Results of Frequency Measurement

For our experiments, the FPGA we used to implement the accelerometer frequency sampling system has a standard frequency of 80 MHz. The measured frequency range is between 20 KHz and 50 KHz, and the gate time is 20 ms. The test results of nine-channel frequency signals of three quartz accelerometers are shown in Table 1. 

From Table 1, we can see the absolute error of the frequency measurement module is less than 0.05 Hz. According to the scale factor of the quartz beam accelerometer, which is 50 Hz/g, the measurement accuracy of the frequency measurement module is less than 1 mg. The long-term cumulative error of the frequency measurement is less than $10^{-4}$. Taking the Y1 channel as an example, the theoretical value measured by *f_x_* is 29,999.9511 Hz. The measured count value, *N_s_*, is 2037335, *N_x_* is 661, and the measured value of *f_x_* calculated by Equation (4) is 29,999.9747 Hz. Therefore, the absolute error of the precision frequency measurement module is 0.0236 Hz, and the relative error is $5.53\times 10^{-7}$.

If the system uses software to calculate the frequency measurement value, the calculation time from sampling to attitude angle compensation is 95.8 µs. To speed up the calculation, a hardware FPU unit is used to compute the frequency measurement value. In the case of using FPU, the calculation time is 27.8 µs. Compared to the software approach, the running time is reduced by 40.8%, and the performance is improved by three times.

### 4.5. Experimental Results of Data Fusion 

**Figure 10:**
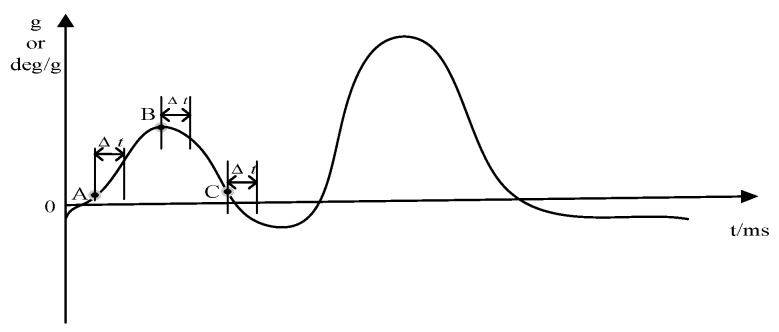
Synchronous and asynchronous acquisition comparison.

The comparison of synchronous and asynchronous acquisition is shown in Figure 10. During the same cycle, suppose that the gyroscope acquires data once every 5 ms, and the sampling points are A, B, and C. Using our proposed synchronous acquisition scheme, the acceleration sampling points are also A, B, and C. However, in the current SINS, the accelerometer and gyroscope work independently under their respective sampling frequencies. There is a degree of asynchrony between them that leads to a certain error, Δt, so asynchronous acquisition will cause a large cumulative error. It has an adverse impact on the accuracy of the final calculated attitude angle and even causes the data to be inaccurate. However, the synchronous acquisition scheme makes the final calculated attitude angle close to the ideal value.

In this paper, we performed high-precision frequency measurement on the QVBA, which improves the accuracy of the acceleration value accordingly. By using the FPU, we accelerated the calculation of the frequency measurement value. It saved conversion time. During the synchronous data acquisition of the accelerometer and the gyroscope, the error caused by the asynchronous degree is wiped out. As the gyroscope integration generates a cumulative error, while the accelerometer compensates it for its drift, thus improving the accuracy of the attitude angle. 

To validate the effect of our proposed scheme, we conducted experimental verification on the simulation platform of IMU. The initial data of QVBA and the gyroscope together with the attitude angle calculated by the simulation platform of IMU were imported into MATLAB for experimental analysis. Taking the pitch angle and the roll angle as an example, the analysis results are shown in the following Figures.

**Figure 11:**
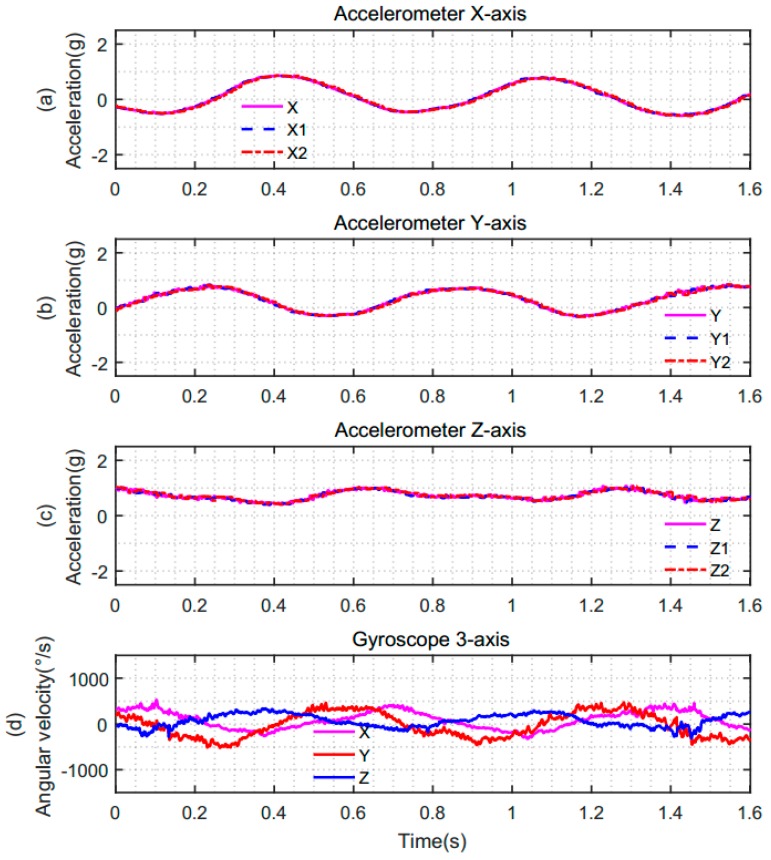
The data of the accelerometer and gyroscope. (**a**) *X*-axis data of the accelerometer; (**b**) *Y*-axis data of the accelerometer; (**c**) *Z*-axis data of the accelerometer; (**d**) three-axis data of the gyroscope.

Figure 11 represents the synchronous and asynchronous sampling data of the accelerometer and gyroscope under the same sampling period. In the case of asynchronous sampling, there is a degree of asynchrony between the accelerometer and gyroscope, which resulted in differences in their sampling instants when the sampling results were output. The sampling data of the accelerometer and gyroscope are shown in Figure 11a–c. The solid pink line indicates the three-axis data of the accelerometer in the case of theoretical synchronization. The blue dashed line indicates the three-axis data of the accelerometer in the case of actual synchronization. The red dotted line indicates the three-axis data of the accelerometer in the case of non-synchronization. Figure 11d shows the three-axis angular velocity data of the gyroscope in the three cases. From this figure, we can see that the data of the gyroscope remains unchanged in the above three different cases. 

**Figure 12:**
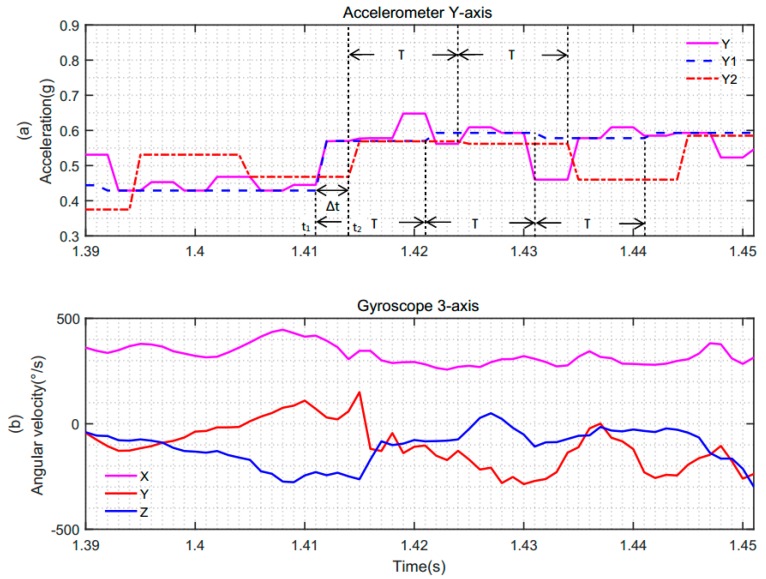
Comparisons of sampling instants of the accelerometer and gyroscope in the case of synchronous and asynchronous sampling. (**a**) *Y*-axis data of the accelerometer; (**b**) three-axis data of the gyroscope.

To further analyze the differences between synchronous sampling and asynchronous sampling, we partially enlarged Figure 11, as shown in Figure 12. Figure 12a represents the synchronous and asynchronous sampling data of the accelerometer. Figure 12b shows the three-axis angular velocity data of the gyroscope. Here, we take the *Y*-axis data of the accelerometer as an example. In Figure 12a, the data of the accelerometer and gyroscope were synchronously acquired at time t1 under the same original sampling period, T, and T was 10ms. However, in an asynchronous system, suppose that the data of the gyroscope are acquired at time t1, the acceleration will be at any position of a sampling calculation period, T. Suppose that the acceleration is sampled at time t2 in Figure 12, there is an error of Δ*t* between the acceleration sample and the gyroscope sample. It accumulates large errors, resulting in inaccurate attitude angles. In this case, the result of the fusion is not accurate. 

**Figure 13:**
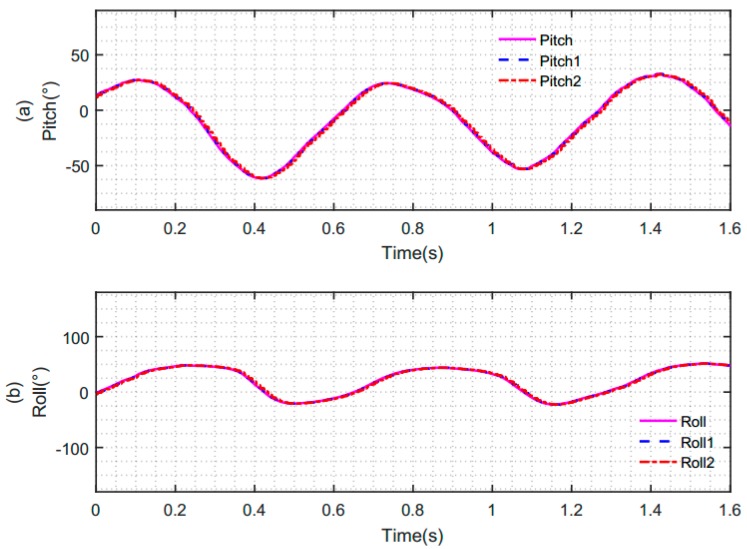
Attitude angle obtained by fusion of the accelerometer and gyroscope data. (**a**) Pitch angle obtained by fusion of the accelerometer and gyroscope under different conditions; (**b**) roll angle obtained by fusion of the accelerometer and gyroscope under different conditions.

In the case of synchronization and non-synchronization above, the initial data of the accelerometer and gyroscope data are fused by a complementary filtering algorithm based on the quaternion method. The obtained attitude angles are as shown in Figure 13. The solid pink line is the pitch angle and roll angle of the attitude angle obtained under the situation of theoretical synchronous data acquisition. The blue dotted line is the pitch angle and roll angle of attitude obtained by our synchronous acquisition mechanism. The red dotted line is the pitch angle and roll angle of attitude obtained under the situation of the non-synchronous data acquisition. 

**Figure 14:**
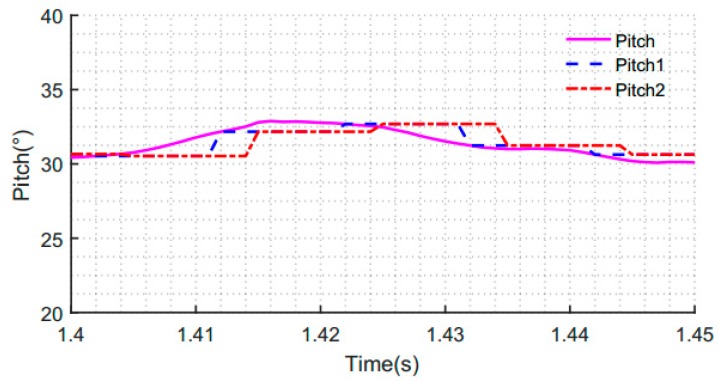
Partially enlarged pitch angle obtained by fusion of the accelerometer and gyroscope data.

To clearly see the effect of the attitude angle obtained in Figure 13, we partially enlarged Figure 13, as shown in Figure 14. Taking the pitch angle as an example, we can see that the blue dotted line is closer to the solid pink line, and the red dotted line is far from the solid pink line. In other words, the attitude angle obtained by using the synchronous acquisition mechanism data is more accurate and more in real-time. In the case of non-synchronization, the obtained attitude angle is not accurate. It is further shown that the degree of synchronization between the acceleration measurement and the attitude angle of the gyroscope has a great influence on the accuracy of the compensation and correction. These experimental results verify the validity of the proposed methods.

**Figure 15:**
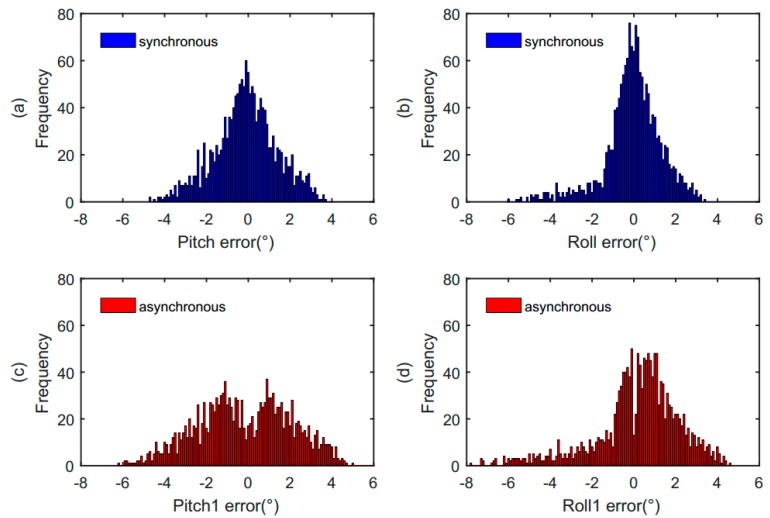
The statistics comparisons of pitch angle error and roll angle error under different conditions. (**a**) The distribution of pitch angle error in the case of synchronization; (**b**) the distribution of roll angle error in the case of synchronization; (**c**) the distribution of pitch angle error in the case of non-synchronization; (**d**) the distribution of roll angle error in the case of non-synchronization.

To evaluate the performance of our proposed solution, we took samples of 1600 values to conduct the verification. We compared two situations, one is to calculate the difference of the attitude angle between synchronous data acquisition and theoretical data acquisition, the other is to calculate the difference of attitude angle between non-synchronous data acquisition and theoretical data acquisition. By the comparison results, it is further verified that the attitude angle obtained by our proposed scheme is closer to the theoretical value. The statistical results of these two comparative experimental data are shown in Figure 15. Figure 15a,b shows the distribution of attitude angle error obtained in the case of synchronization. Figure 15c,d represents the distribution of attitude angle error obtained in the case of non-synchronization. It can be seen from the figures that the distributions of the error data in Figure 15c,d are more dispersed than that in Figure 15a,b. The attitude angle error in the case of synchronization is smaller than that in the case of non-synchronization. The verification results indicate that the attitude angle obtained by our proposed synchronization scheme is more accurate. 

Table 2 is the distribution range of the attitude angle error in the case of synchronization and non-synchronization. The statistical results show that the maximum error of pitch angle is 4.69° in the case of synchronization. However, in the case of non-synchronization, the maximum error of pitch angle is 6.17°. With our proposed synchronization scheme, the pitch angle error is reduced by 24% compared with that of non-synchronization. The maximum error of roll angle is 5.9° in the case of synchronization and is 7.71° in the case of non-synchronization. So, the roll angle error obtained synchronously is reduced by 23.5% compared with that obtained asynchronously. In addition to the maximum error, the sample mean of the attitude angle error in the case of synchronization is reduced by 41.6% compared with the asynchronous one. The experimental results demonstrate that our proposed synchronization scheme is effective in improving the accuracy of the attitude angle.

## 5. Conclusions

In the paper, to improve the attitude angle accuracy of the SINS, a synchronous acquisition scheme to fulfill synchronization of the accelerometer and the gyroscope was proposed. In the process of acceleration change, this method was beneficial to the correction and compensation of the attitude angle. In addition, to improve the performance of QVBAs in the SINS, we proposed an improved equal-precision frequency measurement method, which made the sampling precision of the QVBA more accurate. Furthermore, we introduced a novel FPU to improve the calculation speed of the frequency measurement value. Our proposed methods were verified by experiments. We showed that the accuracy of the attitude angle was improved.

## Figures and Tables

**Table 1:** Test results.

Channel Number	Theoretical Value/kHz	Measured Value/kHz	Absolute Error/Hz
X1	17.9909751	17.9909874	0.0123
X2	14.0023108	14.0023204	0.0096
X3	22.0183216	22.0183232	0.0016
Y1	29.9999511	29.9999747	0.0236
Y2	25.9739942	25.9740014	0.0072
Y3	33.9942926	33.9943092	0.0166
Z1	41.9579906	41.9580023	0.0117
Z2	37.9746063	37.9746556	0.0493
Z3	45.9769553	45.9769680	0.0127

**Table 2:** Test error statistics.

Attitude Angle	Error Range		Sample Mean	
	Synchronous	Asynchronous	Synchronous	Asynchronous
Pitch	[−4.69° 3.78°]	[−6.17° 5.02°]	−0.07°	−0.12°
Roll	[−5.90° 3.51°]	[−7.71° 4.65°]	0.14°	0.24°
